# Comparison of the distribution of non-AIDS Kaposi's sarcoma and non-Hodgkin's lymphoma in Europe

**DOI:** 10.1038/sj.bjc.6690026

**Published:** 1999-09-24

**Authors:** L Dal Maso, S Franceschi, A Lo Re, C La Vecchia

**Affiliations:** Servizio di Epidemiologia, Centro di Riferimento Oncologico, Via Pedemontana Occ.le, Aviano, 12 33081 (PN), Italy; Istituto di Ricerche Farmacologiche ‘M Negri’, via Eritrea 62, Milano 20157, Italy; Istituto di Statistica Medica e Biometria,Uníversità degli Studi, Milano, Italy

**Keywords:** Kaposi's sarcoma, non-Hodgkin's lymphoma, incidence, Europe, immunodeficiency

## Abstract

To evaluate whether some form of mild immunosuppression may influence the geographical distribution of non-AIDS Kaposi's sarcoma (KS), we correlated incidence rates of KS and non-Hodgkin's lymphoma in individuals aged 60 or more in 18 European countries and Israel. Significant positive correlations emerged but, within highest risk countries (i.e.Italy and Israel), internal correlations were inconsistent. © 1999 Cancer Research Campaign


*British Journal of Cancer* (1999) **79**(1), 161–163
© 1999 Cancer Research Campaign

Short communication

### Comparison of the distribution of non-AIDS Kaposi's sarcoma and non-Hodgkin's lymphoma in Europe

**L Dal Maso[1], S Franceschi[1], A Lo Re[1] and C La Vecchia[2,3]**

[1]Servizio di Epidemiologia, Centro di Riferimento Oncologico, Via Pedemontana Occ.le, 12 33081 Aviano (PN), Italy; [2]Istituto di Ricerche Farmacologiche 'M. Negri', via Eritrea, 62, 20157 Milano, Italy; [3]Istituto di Statistica Medica e Biometria, Uníversità degli Studi, Milano, Italy

**Summary** To evaluate whether some form of mild immunosuppression may influence the geographical distribution of non-AIDS Kaposi's sarcoma (KS), we correlated incidence rates of KS and non-Hodgkin's lymphoma in individuals aged 60 or more in 18 European countries and Israel. Significant positive correlations emerged but, within highest risk countries (i.e. Italy and Israel), internal correlations were inconsistent.

**Keywords:** Kaposi's sarcoma; non-Hodgkin's lymphoma; incidence; Europe; immunodeficiency

A mass of epidemiological evidence suggests that Kaposi's sarcoma (KS) is caused by a sexually transmitted infectious agent and is made a thousandfold more frequent by immunodeficiency (Kinlen, 1996; Moore and Chang, 1998). The newly described human herpesvirus 8 (HHV-8) has been detected in nearly all biopsy specimens from patients with all forms of KS (Moore and Chang, 1998).

The lack of an excess of AIDS-associated KS in southern Europe, despite the strong geographical predilection described for classic KS, has long been noticed (Casabona et al, 1991; Ebrahim et al, 1997; Franceschi et al, 1997) and remains unexplained. In fact, the prevalence of KS among AIDS patients in Europe accurately reflects the proportion of homosexual and bisexual men (highest in Denmark, the Netherlands and the United Kingdom) and of people from Africa (highest in Belgium, France and in the United Kingdom) (Dal Maso et al, 1995; Parkin et al, 1997). Even among patients with heterosexually acquired AIDS, however, southern Europe does not show especially high prevalences of KS as an AIDS-defining condition. Italy, for instance, is with Iceland (Hjalgrim et al, 1998) the highest risk country for classic KS (Parkin et al, 1997), but showed in 1981–94 a prevalence of KS as an AIDS-defining condition in Italian-born male heterosexuals of only 7% (IARC, 1996). This proportion is comparable with those seen in north European countries, where classic KS is very rare, e.g. 9% in France, 7% in Germany and 5% in the United Kingdom (IARC, 1996). It has, thus, been suggested that the geographical variations in classic KS may not be due to differences in prevalence of the putative causal virus, but may derive instead from variations in the prevalence of some form of mild immunodeficiency (Ebrahim et al, 1997).

Non-Hodgkin's lymphoma (NHL) incidence, as KS incidence, is greatly increased in immunodeficiency of different origins (Kinlen, 1996). An excess of skin cancer after NHL and an excess of NHL after skin cancer have been reported (Adami et al, 1995; Levi et al, 1996) and attributed to mild immunodepression induced by sunlight exposure (Cartwright et al, 1994). However, data from the United States showed that sunlight did not bear the same clear relationship to all NHL (Hartge et al, 1996; Newton, 1997) or to primary cutaneous NHL (Newton, 1997) that was evident in the geographical variation of melanoma of the skin or non-melanomatous skin cancer. Except for a very rare variety (body cavity-based lymphoma), NHL is not associated with HHV-8 (Cesarman et al, 1995). It is, thus, of interest to examine the most recent (1988–92) incidence rates of non-AIDS KS and NHL in different European countries (Parkin et al, 1997) for evidence of a correlation between these malignancies.

*Received 18 March 1998*
*Revised 12 May 1998*
*Accepted 26 May 1998*

*Correspondence to:* S Franceschi, Servizio di Epidemiologia, Centro di Riferimento Oncologico, Via Pedemontana Occ.le, 12, 33081 Aviano (PN), Italy

### MATERIALS AND METHODS

Age-standardized (world population) incidence rates per 100 000 population in 1988–92 have been derived either for whole nations (e.g. Nordic countries) or for a combination of all available cancer registries (ranging from two for Germany to 13 for Italy) (Parkin et al, 1997). Israel is part of the World Health Organization European Region and was also examined. A few countries (i.e. Austria, Croatia, Ireland, Latvia and Slovenia) for which KS incidence rates were not available (Parkin et al, 1997) have not been considered. Restriction to cancer diagnoses in individuals age 60 years or more should have largely eliminated the confounding influence of AIDS because only about 3% of AIDS cases occur above this age in the countries examined (Dal Maso et al, 1995).

### RESULTS

Standardized incidence rates per 100 000 for KS and NHL in men and women age 60 years or more are given in Table 1 for 19 European countries. KS incidence ranged from 0.1 (Germany and Scotland) to 15.5 (Israel) in men and 0.02 (Slovakia) to 5.5 (Israel) in women. The range for NHL was also substantial: from 13.3 (Belarus) to 59.0 (Switzerland) in men and from 7.0 (Belarus) to 48.0 (Israel) in women.

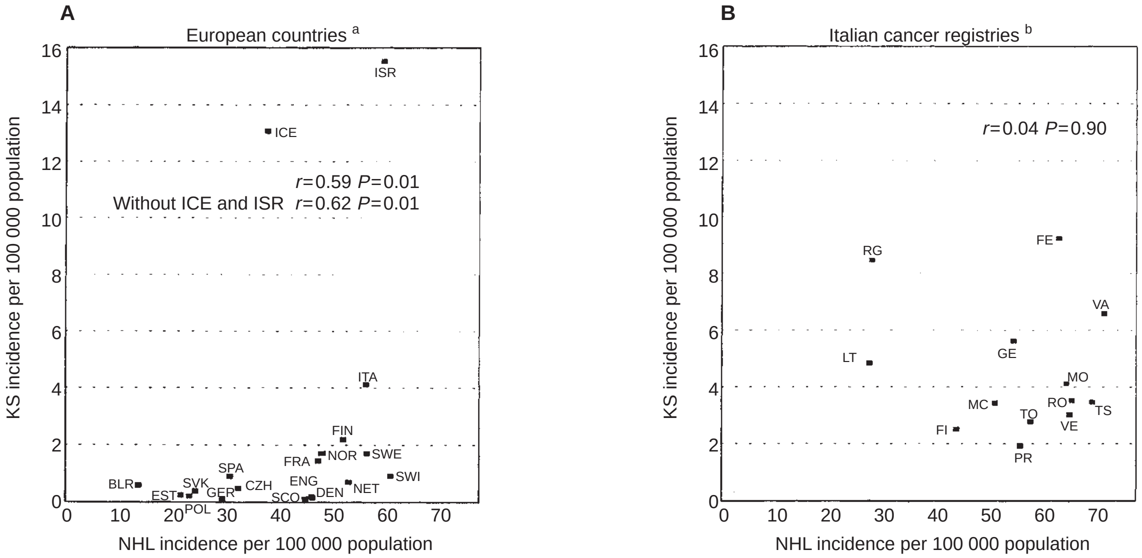

**Figure 1** Correlation between standardized (world) incidence rates per 100 000 population of Kaposi's sarcoma (KS) and non-Hodgkin's lymphoma (NHL) in men age 60 years or more in 19 European countries and in 13 Italian cancer registries, 1988–92 (Parkin et al, 1997). *r* represents the Spearman correlation coefficient. [a]BLR, Belarus; CZH, Czech Republic; DEN, Denmark; ENG, England and Wales; EST, Estonia; FIN, Finland; FRA, France; GER, Germany; ICE, Iceland; ISR, Isreal; ITA, Italy; NET, Netherlands; NOR, Norway; POL, Poland; SCO, Scotland; SPA, Spain; SVK, Slovakia; SWE, Sweden; and SWI, Switzerland. [b]FE, Ferrara; FI, Florence; GE, Genoa; LT, Latina; MC, Macerata; MO, Modena; PR, Parma; RG, Ragusa; RO, Romagna; TO, Torino; TS, Trieste; VA, Varese; and VE, Venetian region

**Table 1** Standardized (world) incidence rates per 100 000 population of Kaposi's sarcoma and non-Hodgkin's lymphoma in men and women age 60 years or more in 19 European countries, 1988–92 (Parkin et al, 1997)

| European countries | Kaposi's sarcoma | | | | Non-Hodgkin's lymphoma | | | |
|---|---|---|---|---|---|---|---|---|
| | Men | | Women | | Men | | Women | |
| | No. | Rate | No. | Rate | No. | Rate | No. | Rate |
| Belarus | 14 | 0.59 | 9 | 0.18 | 358 | 13.28 | 374 | 6.99 |
| Czech Republic | 18 | 0.48 | 11 | 0.18 | 1183 | 31.93 | 1311 | 21.76 |
| Denmark | 4 | 0.16 | 2 | 0.05 | 1092 | 44.89 | 1107 | 32.39 |
| England and Wales[a] | 27 | 0.21 | 11 | 0.05 | 6341 | 44.76 | 6348 | 30.18 |
| Estonia | 1 | 0.25 | 1 | 0.13 | 97 | 21.02 | 95 | 10.53 |
| Finland | 49 | 2.19 | 33 | 0.60 | 1100 | 50.52 | 1508 | 39.67 |
| France[a] | 29 | 1.44 | 8 | 0.26 | 999 | 45.97 | 1099 | 33.37 |
| Germany[a] | 3 | 0.12 | 1 | 0.01 | 739 | 28.54 | 948 | 18.67 |
| Iceland | 13 | 13.09 | 10 | 5.38 | 34 | 36.54 | 26 | 22.40 |
| Israel | 245 | 15.52 | 113 | 5.47 | 824 | 57.69 | 847 | 47.96 |
| Italy[a] | 150 | 4.12 | 71 | 1.09 | 1927 | 54.54 | 1947 | 37.06 |
| Netherlands | 31 | 0.70 | 9 | 0.10 | 2387 | 51.47 | 2382 | 33.98 |
| Norway | 43 | 1.70 | 29 | 0.89 | 981 | 46.47 | 958 | 32.67 |
| Poland[a] | 4 | 0.21 | 2 | 0.07 | 429 | 22.59 | 358 | 11.99 |
| Scotland | 3 | 0.12 | 4 | 0.17 | 970 | 43.62 | 1299 | 38.25 |
| Spain[a] | 27 | 0.90 | 10 | 0.19 | 898 | 29.86 | 913 | 22.83 |
| Slovakia | 6 | 0.38 | 1 | 0.02 | 390 | 23.61 | 376 | 15.90 |
| Sweden | 99 | 1.70 | 42 | 0.52 | 2600 | 54.79 | 2311 | 36.10 |
| Switzerland[a] | 14 | 0.91 | 3 | 0.11 | 913 | 58.99 | 916 | 38.41 |

[a]Rates refer to some areas only.

Positive correlations emerged between KS and NHL in men (Spearman non-parametric correlation coefficient, $r$ = 0.59, $P$ = 0.01) (Figure 1A) and women ($r$ = 0.46, $P$ = 0.05). The exclusion of Iceland and Israel, which could be considered outliers, affected correlation coefficients very little ($r$ = 0.62 in men and 0.44 in women).

However, within the highest-risk countries where regions or groups could be assessed separately, internal correlations did not confirm the pattern suggested above. Among 13 Italian cancer registries, KS and NHL incidence rates were not correlated: Spearman $r$ = 0.04, $P$ = 0.90, in men (Figure 1B) and $r$ = 0.08, $P$ = 0.80, in women. In Israel, where four major groups can be distinguished by Jewish ancestry and place of birth, KS incidence was highest among Jews born in Africa or Asia (25.5 per 100 000 men and 7.6 per 100 000 women). Conversely, NHL incidence was highest among Jews born in Europe or America for men

*© Cancer Research Campaign 1999*

(66.0 per 100 000) and among those born in Israel for women (54.5 per 100 000).

### DISCUSSION

Population-based incidence data have a few important limitations (e.g. lack of distinction between different NHL histological types and sites, and quality problems, particularly among the elderly and in large Eastern European registries; Parkin et al, 1997). These may contribute to the fact that the distribution of classic KS and NHL in European countries provides only partial and inconsistent support to a link between KS and NHL outside AIDS. Israel and Italy show a high incidence of both KS and NHL but Iceland, whose KS excess clearly antedates AIDS (Hjalgrim et al, 1998), has only intermediate NHL rates. The lack of a clear NHL excess in individuals with a previous diagnosis of classic KS (Biggar et al, 1994; Franceschi et al, 1996; Hjalgrim et al, 1997) also weighs against the 'mild immunodeficiency' hypothesis, although larger and better studies should clarify this issue further.

### ACKNOWLEDGEMENTS

This work was supported by two grants from the Ministero della Sanità – Istituto Superiore di Sanità, ISS 1997 contract no. 20.A.11 and ISS 1998 contract no. 20A.0.19. The authors wish to thank Mrs Ilaria Calderan for technical assistance.

### REFERENCES

Adami J, Frisch M, Yuen J, Glimelius B and Melbye M (1995) Evidence of an association between non-Hodgkin's lymphoma and skin cancer. *Br Med J* **310**: 1491–1495

Biggar RJ, Curtis RE, Cotè TR, Rabkin CS and Melbye M (1994) Risk of other cancers following Kaposi's sarcoma: relation to acquired immunodeficiency syndrome. *Am J Epidemiol* **139**: 362–368

Cartwright R, McNally R and Staines A (1994) The increasing incidence of non-Hodgkin's lymphoma (NHL): the possible role of sunlight. *Leukaemia Lymphoma* **14**: 387–394

Casabona J, Melbye M, Biggar RJ and the AIDS Registry Contributors (1991) Kaposi's sarcoma and non-Hodgkin's lymphoma in European AIDS cases. No excess risk of Kaposi's sarcoma in Mediterranean countries. *Int J Cancer* **47**: 49–53

Cesarman E, Chang Y, Moore PS, Said JW and Knowles DM (1995) Kaposi's sarcoma-associated herpesvirus-like DNA sequences in AIDS-related body-cavity-based lymphomas. *N Engl J Med* **332**: 1186–1191

Dal Maso L, Franceschi S, Negri E, Serraino D, La Vecchia C and Ancelle-Park RA (1995) Trends of AIDS incidence in Europe and the United States. *Soz Praeventivmed* **40**: 239–265

Ebrahim SH, Peterman TA, Zaidi AA and Hamers FF (1997) Geography of AIDS-associated Kaposi's sarcoma in Europe. *AIDS* **11**: 1739–1745

Franceschi S, Arniani S, Balzi D and Geddes S (1996) Survival of classic Kaposi's sarcoma and risk of second cancer. *Br J Cancer* **74**: 1812–1814

Franceschi S, Dal Maso L, Lo Re A, Serraino D and La Vecchia C (1997) Trends of Kaposi's sarcoma at AIDS diagnosis in Europe and the United States, 1987–94. *Br J Cancer* **76**: 114–117

Hartge P, Devesa SS, Grauman D, Fears TR and Fraumeni Jr JF (1996) Non-Hodgkin's lymphoma and sunlight. *J Natl Cancer Inst* **88**: 298–300

Hjalgrim H, Frisch M, Pukkala E, Tulinius H, Ekbom A, Dictor M, Langmark F, Hardarson S and Melbye M (1997) Risk of second cancers in classical Kaposi's sarcoma. *Int J Cancer* **73**: 840–843

Hjalgrim H, Tulinius H, Dalberg J, Hardarson S, Frisch M and Melbye M (1998) High incidence of classical Kaposi's sarcoma in Iceland and the Faroe Islands. *Br J Cancer* **77**: 1190–1193

IARC Working Group on the Evaluation of Carcinogenic Risks to Humans (1996) *IARC Monographs on the Evaluation of Carcinogenic Risks to Humans. Human Immunodeficiency Viruses and Human T-cell Lymphotrophic Viruses*, Vol. 67. IARC: Lyon

Kinlen LJ (1996) Immunologic factors, including AIDS. In *Cancer Epidemiology and Prevention*, 2nd edn. Schottenfield D and Fraumeni Jr JF (eds), pp. 532–545. Oxford University Press: New York

Levi F, Randimbison L, Te V-C and La Vecchia C (1996) Non-Hodgkin's lymphomas, chronic lymphocytic leukaemias and skin cancers. *Br J Cancer* **74**: 1847–1850

Moore PS and Chang Y (1998) Kaposi's sarcoma (KS), KS-associated herpesvirus, and the criteria for causality in the age of molecular biology. *Am J Epidemiol*, **147**: 217–221

Newton R (1997) Solar ultraviolet radiation is not a major cause of primary cutaneous non-Hodgkin's lymphoma. *Br Med J* **314**: 1483–1484

Parkin DM, Whelan SL, Ferlay J, Raymond L and Young J (1997) *Cancer Incidence in Five Continents*, Vol. VII. IARC Scientific Publication no. 143. IARC: Lyon

*© Cancer Research Campaign 1999*

